# Peer Review of “Improving Tuberculosis Detection in Chest X-Ray Images Through Transfer Learning and Deep Learning: Comparative Study of Convolutional Neural Network Architectures”

**DOI:** 10.2196/77171

**Published:** 2025-07-01

**Authors:** Rapeepan Pitakaso

**Affiliations:** 1 University of Vienna, remove, Vienna, Austria

**Keywords:** tuberculosis detection, tuberculosis, TB, chest x-ray classification, diagnostic imaging, radiology, medical imaging, convolutional neural networks, data augmentation, deep learning, early warning, early detection, comparative study

*This is a peer-review report for “Improving Tuberculosis Detection in Chest X-Ray Images Through Transfer Learning and Deep Learning: Comparative Study of Convolutional Neural Network Architectures.”*

## Round 1 Review

### General Comments

#### Clarity and Structure

The paper [1] presents a comprehensive overview of the methods and results but can benefit from clearer transitions between sections. For instance, adding brief connecting sentences at the end of each section would help guide the reader into the next topic.

Consider reorganizing the “Discussion” section to first summarize the key findings before delving into their implications. This will reinforce the reader’s understanding of the main outcomes.

#### Writing Style

Aim for more active voice usage to enhance readability. For example, change “It was observed that VGG16 outperformed other models” to “We observed that VGG16 outperformed other models.”

Simplify overly technical or long sentences to improve readability. Breaking complex sentences into two simpler ones can make the content easier to follow.

### Specific Comments by Section

#### Abstract

Sentence clarification: The phrase “necessitating more efficient and accurate diagnostic methods” could be expanded to briefly indicate why current methods are insufficient.

Results detail: When mentioning model performance, briefly state why VGG16’s superior performance is significant compared to others.

#### Introduction

Background information: The explanation of the global tuberculosis burden is informative, but it could benefit from briefly mentioning current limitations in artificial intelligence–based tuberculosis detection in developing countries.

Motivation clarification: Ensure that the motivation for choosing specific convolutional neural network architectures is clearly linked to gaps in existing literature.

#### Methods

Preprocessing details: The detailed explanation of normalization and data augmentation is excellent, but it might be beneficial to briefly mention how these choices align with previous research findings or unique aspects of this study.

Transfer learning: Include a brief comparison of why transfer learning was chosen over training models from scratch.

#### Results

Visualization: The table summarizing model performance is comprehensive, but consider including a concise narrative to describe key trends observed in the data.

Analysis clarification: When discussing why data augmentation did not enhance performance, elaborate on how this aligns with or contradicts findings from other studies.

#### Discussion

Comparison with previous studies: Add a few sentences comparing the results with existing studies that used the same models or datasets to provide context.

Implications: Discuss the practical implications of using VGG16 in resource-constrained environments where computational efficiency is crucial.

#### Conclusion

Highlight novelty: Emphasize what makes this study’s approach unique, such as the use of specific architectures on a larger dataset, and how this adds to the current body of knowledge.

Future work suggestions: Include more detailed recommendations for future studies, potentially suggesting how to further leverage data augmentation strategies.

### Grammar and Language

Sentence revisions: original: “It is observed that the VGG16 consistently performed better than other models.” Revised: “We observed that VGG16 consistently performed better than the other models.”

Punctuation: Ensure commas are consistently used after introductory phrases (eg, “In this study, we propose...”).

Word choice: Replace terms like “aimed to assess” with “assessed” to make sentences more concise.

### Technical Aspects

Hyperparameter details: Include a brief rationale for choosing the specific hyperparameters in Table 1 to enhance the reader’s understanding.

Training environment: Specify why the computational setup (eg, graphics processing unit details) was chosen and how it impacted training efficiency.

### Final Suggestions

Proofreading: Ensure that each section is proofread for minor grammatical errors or inconsistencies.

Figures and tables: Verify that all figures and tables have descriptive captions, and refer to them within the text to maintain flow.
